# Green Synthesis of *Blumea balsamifera* Oil Nanoemulsions Stabilized by Natural Emulsifiers and Its Effect on Wound Healing

**DOI:** 10.3390/molecules29091994

**Published:** 2024-04-26

**Authors:** Lingfeng Du, Chunfang Ma, Bingnan Liu, Wei Liu, Yue Zhu, Zuhua Wang, Teng Chen, Luqi Huang, Yuxin Pang

**Affiliations:** 1 College of Chinese Medicine Resources, Guangdong Pharmaceutical University, Yunfu 527325, China; dlingf00@163.com (L.D.); m16653194486@163.com (C.M.); lbn0714@163.com (B.L.); l_w_biophilia@163.com (W.L.); 2 College of Pharmaceutical Sciences, Guizhou University of Traditional Chinese Medicine, Guiyang 550025, China; moonzy0210@163.com (Y.Z.); wangrui551601@163.com (Z.W.); 3 Nano-Drug Technology Research Center, Guizhou University of Traditional Chinese Medicine, Guiyang 550025, China; 4 China Academy of Chinese Medical Sciences, Beijing 100700, China; 5 Yunfu Traditional Chinese Medicine Resources and Germplasm Resources Database Management Center, Yunfu 527325, China

**Keywords:** *Blumea balsamifera* oil, nanoemulsions, natural emulsifier, wound healing, network pharmacology

## Abstract

In this study, we developed a green and multifunctional bioactive nanoemulsion (BBG-NEs) of *Blumea balsamifera* oil using *Bletilla striata* polysaccharide (BSP) and glycyrrhizic acid (GA) as natural emulsifiers. The process parameters were optimized using particle size, PDI, and zeta potential as evaluation parameters. The physicochemical properties, stability, transdermal properties, and bioactivities of the BBG-NEs under optimal operating conditions were investigated. Finally, network pharmacology and molecular docking were used to elucidate the potential molecular mechanism underlying its wound-healing properties. After parameter optimization, BBG-NEs exhibited excellent stability and demonstrated favorable in vitro transdermal properties. Furthermore, it displayed enhanced antioxidant and wound-healing effects. SD rats wound-healing experiments demonstrated improved scab formation and accelerated healing in the BBG-NE treatment relative to BBO and emulsifier groups. Pharmacological network analyses showed that AKT1, CXCL8, and EGFR may be key targets of BBG-NEs in wound repair. The results of a scratch assay and Western blotting assay also demonstrated that BBG-NEs could effectively promote cell migration and inhibit inflammatory responses. These results indicate the potential of the developed BBG-NEs for antioxidant and skin wound applications, expanding the utility of natural emulsifiers. Meanwhile, this study provided a preliminary explanation of the potential mechanism of BBG-NEs to promote wound healing through network pharmacology and molecular docking, which provided a basis for the mechanistic study of green multifunctional nanoemulsions.

## 1. Introduction

*Blumea balsamifera* (L.) DC. is a perennial herb or subshrub belonging to the Asteraceae family [1]. It has a long history of medicinal use in Southeast Asian countries, such as China, Thailand, and the Philippines. In China, *Blumea balsamifera* (L.) DC. was first documented in the “Bei Ji Qian Jin Yao Fang” by Sun Simiao in 652. Minority group such as Miao, Li, and Zhuang often use it to treat colds, snakebites, trauma, and burns. As one of its main active ingredients, *Blumea balsamifera* essential oil (BBO) demonstrates multiple pharmacological activities including analgesic, antioxidant, anti-inflammatory, and wound-healing properties [2,3,4,5]. However, as a plant essential oil, BBO has high hydrophobicity and strong volatility, which results in its short duration of efficacy and uncontrollable dosage, making it difficult to apply in practical applications. To address these issues, an effective strategy is to maintain BBO’s performance and enhance its water solubility through incorporation into nanomedicine delivery systems. Among these, oil-in-water (O/W), nanoemulsions (NEs) have proven to be an effective delivery system for loading plant essential oils. O/W NEs have been employed to encapsulate various essential oils, such as *C. cassia* [6] and clove [7]. This process can improve the stability of essential oil and enhance its antioxidant activity or antibacterial ability.

O/W NEs represent a type of nanostructured colloidal system composed of oil droplets dispersed in a continuous aqueous phase, stabilized by surfactant molecules [8]. In comparison with traditional topical formulations, NEs have been shown to have advantages including improved drug solubility, effective protection and transport of insoluble active ingredients, reduced irritation, increased skin contact, and enhanced drug penetration [9,10]. These properties have led to their increasing use in drug delivery applications, particularly for topical medications. NEs are typically unstable systems, prone to issues such as flocculation and phase separation, necessitating the use of emulsifiers to enhance stability. Emulsifiers commonly utilized fall into two categories: synthetic and natural. Studies have shown that synthetic emulsifiers have chronic toxicity and a series of inevitable biological safety hazards [11,12]. In contrast, naturally derived emulsifiers offer favorable attributes such as biodegradability, biocompatibility, and biosafety, making them an active area of investigation in recent years.

Natural emulsifiers mainly include proteins, polysaccharides, saponins, phospholipids, etc. Polysaccharides, as natural polymers, can create a dense hydrophilic layer encircling oil droplets, generating robust repulsion in three dimensions. This leads to effective stabilization of oil–water and air–water interfaces, making polysaccharides a common choice as an emulsifier for NEs [13]. Moreover, polysaccharides possess pharmacological properties, such as antioxidant, antiviral or anti-inflammatory effects, which not only stabilize NEs but also enhance their biological activity. *Bletilla striata* polysaccharide (BSP) is the main active compound in the perennial herb bulbous plant *Bletilla striata* (Thunb.) Reichb. f. (*Bletilla striata*) [14]. Its skeleton is mainly composed of 1,2- or 1,4-linked mannose residues and 1,4-linked glucose residues [15]. BSP possesses diverse pharmacological activities, such as antioxidant, anti-inflammatory, hemostatic, and wound-healing effects [16,17,18,19]. Glycyrrhizic acid (GA), a saponin component, consists of hydrophobic triterpenoid saponin and hydrophilic diglucuronic unit [20]. It is amphiphilic and has the ability to reduce interfacial tension, stabilize the oil–water interface, and form micelles in aqueous solutions to solubilize insoluble components. Consequently, it can serves as an emulsifier for O/W NEs [21]. Furthermore, GA exhibits diverse pharmacological activities like antioxidant, anti-inflammatory, hepatoprotective, and anti-apoptosis effects [22,23,24]. The utilization of natural compounds like BSP and GA as emulsifiers for NEs can enhance stability and reduce excipient toxicity. Furthermore, these compounds exhibit diverse effects, such as antioxidant and anti-inflammatory properties, enabling a synergistic “medicine and excipients all-in-one” function [25]. This ultimately boosts the pharmacological activity of NEs.

The preparation methods of NE include the low-energy method and high-energy method. The low-energy method requires less energy, but usually requires the addition of a large amount of synthetic emulsifiers or co-emulsifiers to stabilize the droplets. The high-energy method uses mechanical force to disperse large emulsion droplets into small ones, resulting in nanoscale emulsion [26]. Compared with the low-energy method, the high-energy method has no restrictions on the types of oil and surfactant, and typically requires lower emulsifier-to-oil ratios than do low-energy methods [27]. High-energy methods include microfluidization, high-pressure homogenization, and ultrasonication (US). US is the use of ultrasonic waves to make the system of incompatible crushed and dispersed liquids homogeneous, and to cause the surrounding liquid to form an emulsion. During the ultrasonic process, a large number of bubbles are generated in the liquid. These small bubbles undergo gradual growth and enlargement due to ultrasonic vibrations, followed by sudden splitting and fragmentation. The subsequent growth and implosive collapse of these fragmented bubbles generate a powerful cavitation effect. This strong cavitation effect initiates the emulsification process of the physical transformation required to produce NEs with droplet sizes less than 400 nm [28,29,30]. Compared to other methods, NEs prepared through US possess small particle sizes, exhibit stable emulsification systems, and require less power [31]. Many studies have demonstrated that US-assisted NEs are efficacious for the encapsulation and delivery of various pharmaceutical and food actives.

Network pharmacology and molecular docking are innovative technologies that integrate multiple disciplines. Network pharmacology involves virtual computing, high-throughput data analysis, network database searching, bioinformatic network construction, and network topology analysis. This method can expose the multi-component, multi-channel, and multi-target synergistic effects, so it is an effective method that can be used as an effective method to study the complex relationship between multi-component drugs and diseases. Molecular docking is a computer simulation technique that can model the interactions between receptors and drugs at the atomic level for drug design, thus predicting their conformations and affinities, etc. In recent years, with the development of bioinformatics, network pharmacology and molecular docking based on computational prediction has become a powerful method to systematically reveal the biological mechanisms and drug effects of complex diseases at the molecular level.

**Scheme 1:**
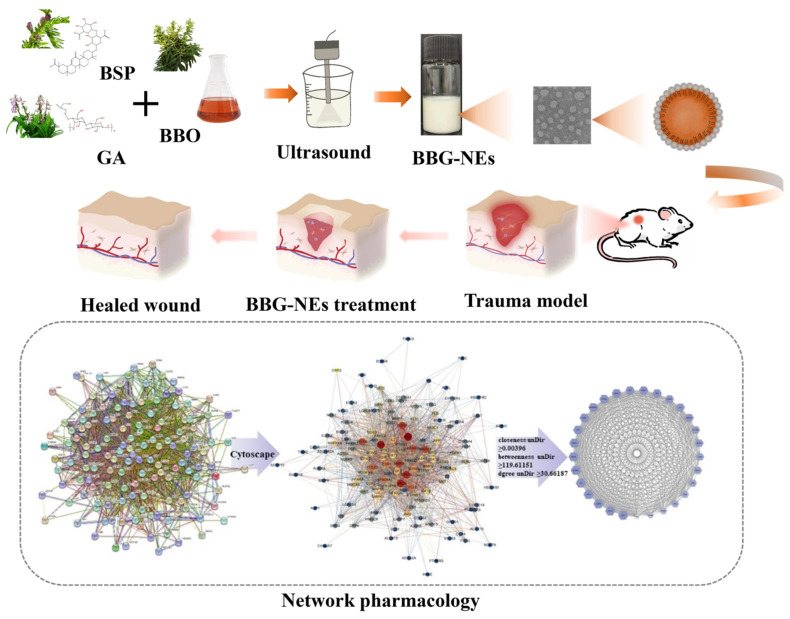
Schematic diagrams of preparation and application of BBG-NEs in wound healing.

In this study, using BBO as the oil phase, we explored the feasibility of the US method for the preparation of BBG-Nes with multiple bioactivities by utilizing natural emulsifiers BSP and GA. The effects of parameters such as oil content, BSP content, and ultrasonic conditions (ultrasonic time and ultrasonic power) on the properties of the NEs were investigated. The stability of the BBG-NEs prepared under optimal conditions was evaluated at different centrifugation speeds and temperatures. The antioxidant activity and pro-wound-healing properties of the BBG-NEs were further investigated, and the mechanism of BBG-NEs for wound healing was explored using network pharmacology and molecular docking. The network pharmacology results were also validated using scratch and Western blotting experiments (Scheme 1). Our findings offer novel insights into the development of multifunctional, bioactive green NEs via US, with potential implications for the biomedical and pharmaceutical sectors.

## 2. Results and Discussion

### 2.1. Optimization Study

Previous studies have indicated that the particle size, PDI, and zeta-potential of NEs are influenced by the oil phase content, emulsifier content, as well as ultrasonic conditions (including ultrasonic time and power) [32]. The smaller the size and narrower the PDI of NEs, the more stable they are. Meanwhile, NEs droplets typically possess a surface potential, known as zeta potential, due to adhesion of charged molecules. Zeta potential plays a significant role in NEs system stability as it contributes to the generation of repulsive electric forces among dispersed phase droplets, hindering the merging process [33,34]. An elevated absolute value of zeta potential signifies better system stability. Generally, a zeta potential absolute value greater than 30 mV is regarded as a suitable indicator for colloidal suspension stability [35,36]. To obtain stable BBG-NEs, single-factor experiments were performed to optimize the above parameters.

**Figure 1:**
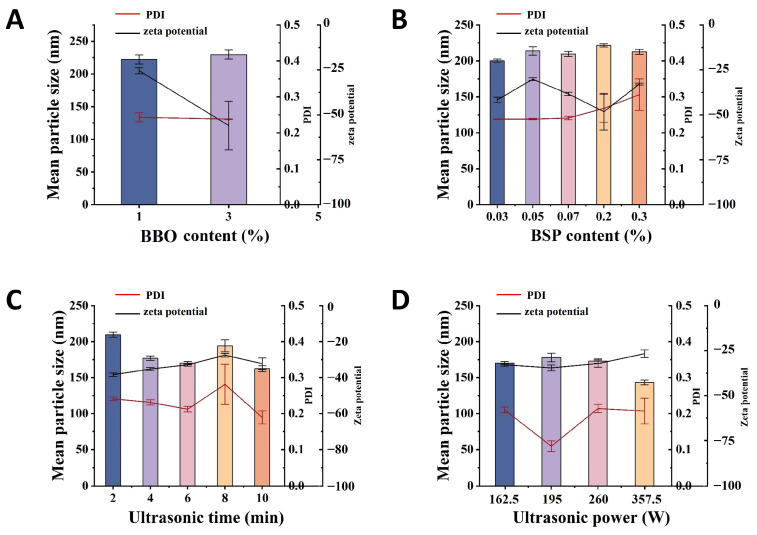
Influence of content of BBO (**A**), content of BSP (**B**), ultrasonic time (**C**), and ultrasonic power (**D**) on the changes in mean particle size, PDI, and zeta potential of the BBG-NEs using US.

The results revealed that when the BBO content exceeds 3% (e.g., 5%, 7%, 10%), the NEs become unstable, leading to phase separation and the presence of floating oil beads (Figure S1 and Table S1). In contrast, stable NEs with a homogeneous milky white appearance were produced at BBO levels of 1% and 3%. The particle size and PDI of these two samples were close, but the absolute value of zeta potential was larger at a BBO percentage of 3% (Figure 1A), and therefore, a BBO content of 3% was selected.

The effect of BSP content on the properties of BBG-NEs is shown in Figure 1B and Table S2. When the concentration of BSP was between 0.03% and 0.30%, the prepared NEs were all homogeneous milky white liquids (Figure S2). The particle size of the NEs remained relatively constant at around 200 nm, and the zeta potential values were all less than −30 mV. However, the PDI started to increase when the BSP content reached 0.20% and 0.3%. At a BSP content of 0.3%, the PDI exceeded 0.3. The PDI value indicates the uniformity of the particle size distribution in the NEs. A smaller PDI indicates better uniformity and easier maintenance of stability in the system. Considering the antioxidant, anti-inflammatory, and hemostatic effects of BSP, the BSP content was determined to be 0.07%.

When the ultrasonic time was between 2 min and 10 min, the prepared NEs were all homogeneous milky white liquids (Figure S3). Figure 1C and Table S3 show the influence of ultrasonic time on particle size, PDI, and zeta potential. The NEs exhibit larger particle size and PDI when the ultrasonic time is short (2 min) due to an inadequate ultrasound process and uneven dispersion. As the ultrasonic time increased from 2 min to 6 min, the particle size and PDI initially decreased and slightly fluctuated subsequently for 10 min. Meanwhile, the zeta potential first increased and then decreased with a longer ultrasonic time. At an ultrasonic time of 8 min, the zeta potential surpassed −30 mV, while for all other ultrasonic times, it remained below −30 mV. A longer ultrasonic time promotes the integration of water and oil. On the other hand, an extended ultrasonic time may cause the degradation or decomposition of bioactive chemicals in BBO, as well as energy wastage [37]. Considering the particle size, PDI, zeta potential, and BBO stability, the optimal ultrasonic time was determined to be 6 min.

To investigate the impact of ultrasonic power on the properties of BBG-NEs, various ultrasonic powers ranging from 65.0 to 357.5 W were used during the preparation process. At an ultrasonic power of 65 W, stable NE formation was not achieved, characterized by phase separation and the presence of floating oil droplets in the liquid (Figure S4). As shown in Figure 1D and Table S4, in the ultrasonic power range of 162.5–260.0 W, the particle size, PDI, and zeta potential of BBG-NEs did not change significantly. While the smallest particle size was observed at 357.5 W, excessive ultrasonic power may compromise the stability of volatile oil in the NEs [36], potentially leading to BBO loss. Therefore, to prevent such loss, an ultrasonic power of 162.5 W was chosen.

### 2.2. Physicochemical Characterizations of BBG-NEs

**Figure 2:**
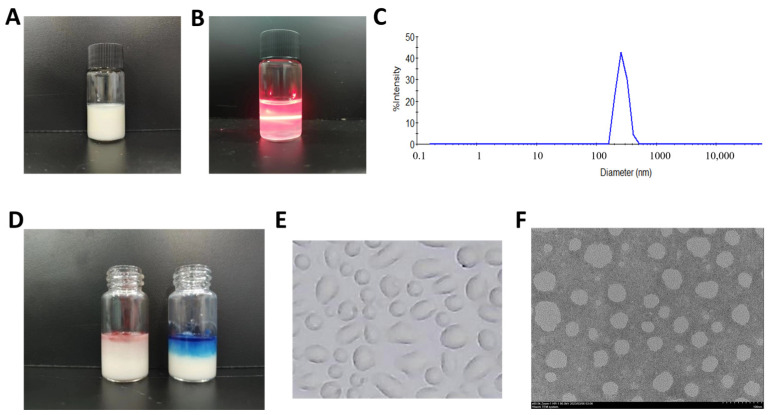
Physicochemical characterization of BBG-NEs: (**A**) appearance of BBG-NEs; (**B**) Faraday–Tyndall effect after 1000-time dilution; (**C**) particle size distribution diagram; (**D**) type identification; (**E**) optical microscope image; (**F**) TEM image.

Based on the results of single factor experiments, BBG-NEs were prepared under the optimal conditions, and their physicochemical characterization and stability were investigated. As shown in Figure 2A, BBG-NEs appeared as a homogeneous milky white appearance. After the dilution of the BBG-NEs, an obvious Faraday–Tyndall effect can be seen, confirming the presence of nanoparticles (Figure 2B). The average particle size and PDI of BBG-NEs was 170.2 ± 2.39 nm (Figure 2C), and 0.212 ± 0.008. Emulsions having particle sizes ranging from 20 to 200 nm exhibit higher stability compared to conventional emulsions and can improve the permeability of encapsulated drugs [38]. Meanwhile, a PDI below 0.3 indicates excellent dispersion of droplets with a narrow size range [39]. The zeta potential of BBG-NEs was −32.86 ± 0.71 mV, which further indicates its excellent stability. To identify the emulsion type, Sudan red (oil soluble) dye and methyl blue (water soluble) dye were added into BBG-NEs, respectively, and their diffusion rates were assessed. As shown in Figure 2D, the diffusion of methyl blue surpasses that of Sudan red, it suggests that the NEs are of the O/W type [40]. The optical microscope and TEM images revealed that BBG-NEs exhibited a uniform spherical morphology, excellent dispersion, and no aggregation (Figure 2E,F). The BBG-NEs exhibited an acidic pH of 3.33, fulfilling the criteria for weak acidity as required in skin preparations.

### 2.3. Stability Studies

#### 2.3.1. Long-Term Stability

**Figure 3:**
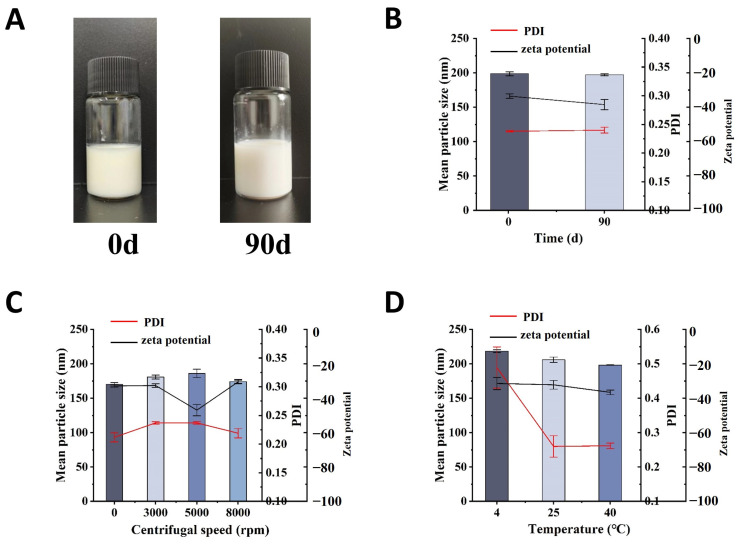
The stability studies: (**A**) appearance of nano-emulsions in long-term stability; (**B**) long-term stability; (**C**) centrifugal stability; (**D**) temperature stability.

The long-term stability of BBG-NEs was evaluated by monitoring the appearance, mean particle size, PDI, and zeta potential at room temperature on day 0 and day 90. No significant changes in appearance or morphology were observed after 90 days, and there were no signs of aggregation, coagulation, phase separation, or emulsion breakage (Figure 3A). Additionally, the mean particle size and PDI of BBG-NEs remained close to their initial values on day 0, while the zeta potential slightly decreased (Figure 3B). These findings indicate that BBG-NEs maintain stability at room temperature for more than 90 days.

#### 2.3.2. Centrifugal Stability

Due to their thermodynamic instability, NEs can exhibit a creamy or detached appearance during long-term storage. Hence, the stability of newly prepared polymer NEs can be assessed by subjecting them to stress conditions to accelerate emulsion rupture, such as centrifugation [41]. After centrifugation at 3000 rpm, 5000 rpm, and 8000 rpm for 10 min, no phase separation, emulsion formation, or precipitation was observed in BBG-NEs (Figure S5). Meanwhile, there were no significant changes in the average particle size, PDI, and zeta potential (Figure 3C).

#### 2.3.3. Temperature Stability

To investigate the impact of temperature on BBG-NEs stability, BBG-NE was placed at 4 °C, 25 °C, and 40 °C for 48 h, respectively. Following this duration, the emulsions maintained a milky, opaque, and uniform appearance at all tested temperatures, without exhibiting precipitation or stratification (Figure S6). Compared with 25 °C, the PDI of BBG-NEs did not change much after 48 h at 40 °C, but the particle size increased to about 200 nm. Meanwhile, at 4 °C, there is an increase in both particle size and PDI, with the latter exceeding 0.4 (Figure 3D). These suggest that BBG-NEs is unstable at 4 °C and 40 °C, which should be stored at room temperature. The zeta potential of BBG-NEs remains unaffected by temperature variations.

### 2.4. In Vitro Skin Permeation Study

**Figure 4:**
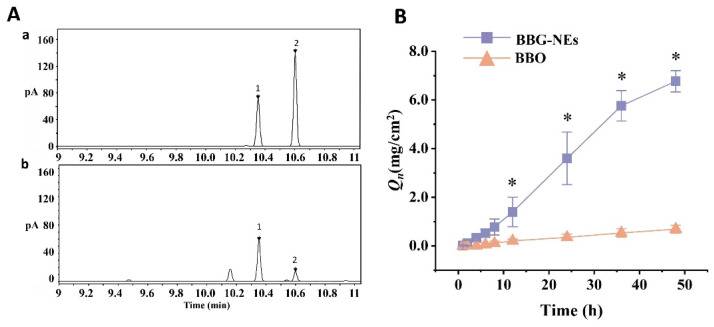
Determination of L-borneol in BBO: (**A**) gas chromatograms of BBG-NEs: (**a**) L-borneol and methyl salicylate; (**b**) BBG-NEs (1: L-borneol; 2: methyl salicylate); (**B**) cumulative permeation of L-borneol in BBG-NEs and BBO through rat skin in vitro. (Data are expressed as mean ± SEM, *n* = 3. Comparisons with individual time points of the BBO release curve are shown; * indicates *p* ≤ 0.05).

To evaluate the in vitro skin permeation of BBG-NEs, L-borneol, the key active component of BBO, was utilized as the test index (Figure 4A). Meanwhile, 3% BBO was employed as the control. As depicted in Figure 4B, the *Q_n_* of BBG-NEs was notably greater than that of BBO group. At 48 h, the *Q_n_* of BBO was 0.69 ± 0.16 mg/cm^2^, with a release rate of only 7.27 ± 1.71%. In contrast, the cumulative release of L-borneol was significantly elevated when prepared as a NE. At 48 h, BBG-NE’s *Q_n_* was 6.77 ± 0.44 mg, which was 9.8 times higher than BBO. These results demonstrated that NEs significantly enhanced the skin penetration of BBO’s active ingredients.

To uncover the release mechanism of BBG-NEs, the in vitro permeation profile was matched to zero-order, first-order, Higuchi, and Ritger–Peppas models. As listed in Table 1, the permeation pattern of BBG-NEs demonstrated the best fit with the zero-order model, displaying the highest r value (r = 0.994) (Figure S7). This suggests that L-borneol was released slowly at a constant rate, independently of the initial drug concentration in the vehicle. In addition, the zero-order model could reduce potential peak/trough fluctuations and side effects, while also prolonging the time the drug concentration remains within the therapeutic window [42].

### 2.5. Antioxidant Capacity

#### 2.5.1. DPPH Free Radical Scavenging Capacity

**Figure 5:**
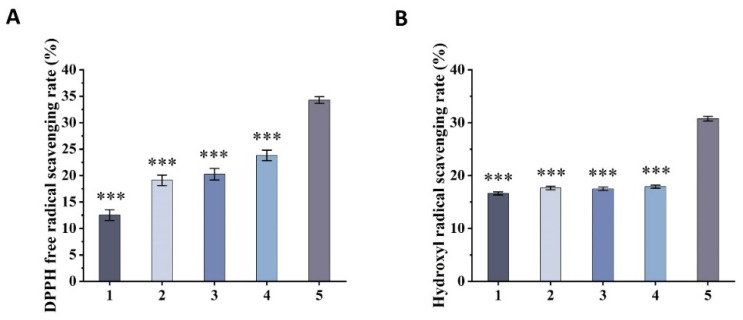
Antioxidant capacity of BBG-NEs: (**A**) DPPH radical scavenging capacity; (**B**) OH radical scavenging capacity (1. GA; 2. BSP; 3. GA + BSP; 4. BBO; 5. BBG-Nes). (Data are expressed as mean ± SEM, *n* = 3. *** indicates *p* ≤ 0.001 compared with the BBG-NEs group).

DPPH radical is a very stable nitrogen-centered radical, which is an important indicator of the antioxidant capacity of the sample. When antioxidants are present, DPPH radicals with single electron are scavenged, the DPPH solution becomes lighter from the purple color, and the absorbance at 515 nm decreases, while the ability of the sample to clear DPPH radicals is reflected by the degree of decrease in absorbance [43]. As depicted in Figure 5A, GA, BSP, mixed emulsifier (GA + BSP), and naked BBO all exhibited DPPH scavenging ability. The DPPH scavenging capacity of mixed emulsifiers is higher than that of individual emulsifiers BSP or GA, whereas the DPPH scavenging capacity of BBG-NEs showed a significantly higher value compared to naked BBO and mixed emulsifiers (GA + BSP). These findings suggest that employing antioxidant activity GA and BSP as emulsifiers in the preparation of BBG-NEs can enhance their DPPH scavenging ability.

#### 2.5.2. Hydroxyl Radical Scavenging Capacity

Hydroxyl radicals are a kind of free radicals produced by the human body in the process of metabolism, and are highly toxic and harmful to living organisms. They can cause oxidative damage to sugars, amino acids, proteins, nucleic acids, and other tissue substances, leading to cell death or mutation. The scavenging capacity of hydroxyl radicals serves as a significant indicator of sample’s antioxidant potential, commonly utilized in the study of antioxidant nutraceuticals and pharmaceuticals [44]. The generation of hydroxyl radicals by H_2_O_2_/Fe^2+^ through the Fenton reaction oxidizes the Fe^2+^ in o-diazo phenanthrene-Fe^2+^ aqueous solution to Fe^3+^, resulting in a decrease in the absorbance of the sample at 536 nm. The degree of inhibition of the rate of decrease in absorbance reflects the ability of the sample to remove hydroxyl radicals [45]. The scavenging rates of GA, BSP, mixed emulsifier (GA + BSP), and BBO for hydroxyl radicals exhibit closely similar values. In contrast, the scavenging rate of BBG-NEs for hydroxyl radicals is significantly higher than that of the above four groups (Figure 5B).

The above findings suggest that the preparation of BBG-NEs with natural emulsifiers GA and BSP with antioxidant activity can significantly enhance its antioxidant activity. Possible reasons for this phenomenon include the following: (1) There may be a synergistic effect between BBO, GA, and BSP in the NEs complex. (2) Pure BBO is insoluble in water, hindering its antioxidant activity. O/W NEs, with their good water solubility and large surface area, facilitate effective delivery of active ingredients and enhance antioxidant capacity [46].

### 2.6. Cytotoxicity Assays

**Figure 6:**
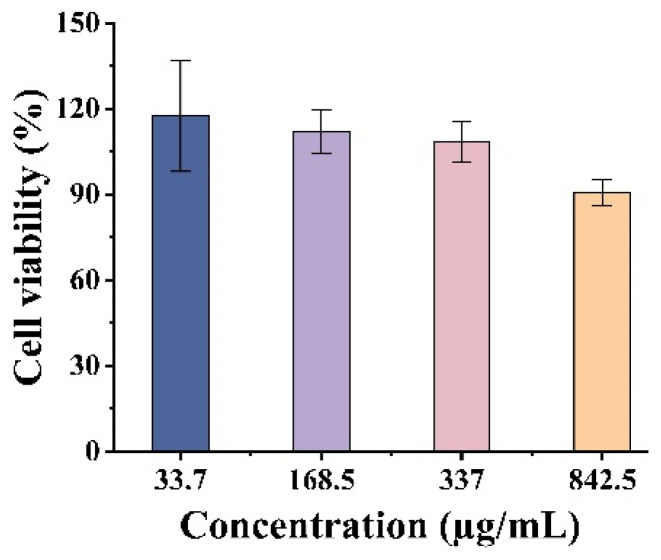
Cytotoxic property of BBG-NEs against L929 cells.

The cytotoxic effects of BBG-NEs were evaluated using the MTT (thiazolyl blue) colorimetric assay. In this assay, succinate dehydrogenase within the mitochondria of cells reduces MTT to insoluble blue-violet Formazan crystals, which are retained by living cells but not by dead ones. Dimethyl sulfoxide (DMSO) is used to solubilize the Formazan within the cells, and the resulting absorbance is used to indirectly quantify the number of viable cells. The amount of Formazan crystals formed is directly proportional to the number of cells within a specific range. Figure 6 illustrates that the cell viability of fibroblast L929 remained above 90% when the concentration of BBG-NEs was between 33.7 and 842.5 µg/mL. These findings suggest that BBG-NEs demonstrate a high level of biosafety towards L929 cells.

### 2.7. Wound-Healing Assessment

**Figure 7:**
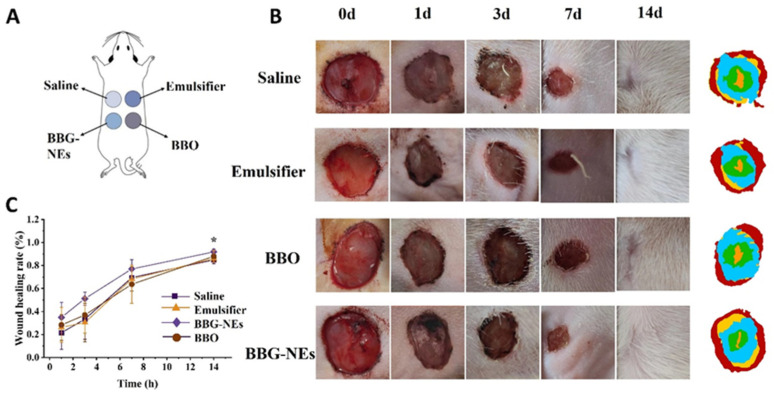
Wound-healing experiment: (**A**) schematic diagram of model construction; (**B**) wound-healing appearance on day 0, 1, 3, 7, and 14; (**C**) wound-healing rate curve. (Data are expressed as mean ± SEM, *n* = 6. * *p* < 0.05 compared with the BC group).

In ancient China, ethnic minorities such as Miao and Zhuang people used fresh ground or mashed juice of *Blumea balsamifera* (L.) DC., *Bletilla striata*, or *Glycyrrhiza glabra* L. (Liquorice) to treat wounds. This traditional method resulted in fast healing without scarring, demonstrating a miraculous effect without advanced medical technology. To assess the healing effects, the rat wound model was used to investigate the impact of naked BBO, emulsifier (GA + BSP), and BBG-NEs. Saline was used as the control group (Figure 7A). The wound-healing progress was monitored daily, and the healing rate was calculated through photographs taken on the 1st, 3rd, 7th, and 14th day. As depicted in Figure 7B, wounds in all groups started shrinking and forming crusts on the first day. By day three, BBG-NEs group wounds were fully crusted and significantly smaller than those of the control, BBO, and emulsifier groups. On day seven, crusts fell off and rapid healing began for both BBG-NEs and emulsifier groups. By day 14, the BBG-NEs group displayed superior recovery, with hair growth around the wounds happening at an accelerated pace, resulting in almost negligible scarring, whereas the saline group wounds remained visible and had less surrounding hair growth. According to Figure 7C, the wound-healing rate of the BBG-NEs group (51.20%) was significant higher that of saline group (33.36%) on day 3. By day 14, the BBG-NEs group had a wound-healing rate of 91.8%, surpassing the rates of the control (84.9%), BBO (87.9%), and emulsifier (86.0%) groups. Although the difference in healing rate between the BBG-NEs and saline groups decreased by day 14, this may be attributed to the longer observation period allowing the wounds in the saline group to also heal. These findings suggest that the synergistic mixture of BBO, BSP, and GA in preparing nano-emulsion can effectively accelerate wound healing.

### 2.8. Network Pharmacology

#### 2.8.1. Screening of Active Ingredients

As a common herb used by the Miao ethnic minority in Guizhou province, *Blumea balsamifera* DC. is currently not included in the TCMSP database. Therefore, the main active components were first obtained using a literature search method. After searching, screening, and de-duplication, 176 components of BBO were obtained [4,47,48,49,50]. Then, SwissADME was used to screen the components of BBO for pharmacodynamic and pharmacokinetic predictions, resulting in 87 active ingredients. In addition, it has been reported in that BSP is composed of mannose and glucose [51,52]. Subsequently, we imported the 87 active ingredients found above in BBO and the two monosaccharides of BSP, along with GA into the SwissTargetPrediction database and excluded the overlapping genes. Finally, 521 associated target genes were identified.

#### 2.8.2. Wound-Healing-Related Target Screening and PPI Network Construction

**Figure 8:**
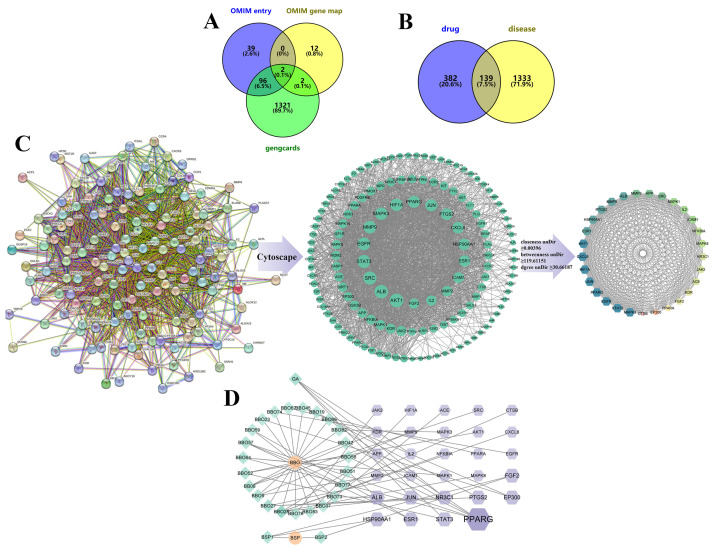
Core targets screening and PPI diagrams: (**A**) Venny diagram of wound-healing targets from different databases; (**B**) Venny diagram of drug ingredients and trauma repair targets; (**C**) PPI diagrams of targets: string establishes a PPI network, imports cytoscape and then goes through centiscape screening to obtain the core target; (**D**) drug–component–target network, with green diamond-shaped nodes representing ingredients in the formulation, purple hexagonal nodes representing trauma repair-related targets, and orange circular nodes representing the drugs. The larger the area of the node graph and the darker the color, the larger the DEGREE value of the component.

The Genecards database suggested that 7319 genes might be linked to wound healing. Genes with a correlation score greater than 2.0 were selected, yielding 1421 genes. Online Mendelian Inheritance in Man (OMIM) also identified 153 genes that could be possibly associated with wound healing. After removing overlapping genes, 1472 wound-healing-related genes were obtained (Figure 8A). Venny 2.1.0 mapped 521 BBG-NEs targeted genes to 1472 wound-healing-related genes, resulting in 139 intersection targets (Figure 8B). Then, centiscape plug-in of Cytoscape 3.9.1 was employed to further perform gene screening, resulting in 29 key intersection targets for BBG-NEs and wound healing were identified (Figure 8C). The above 29 intersectional targets were mapped into a “drug–component–target” interactive network of BBG-NEs regulating wound healing (Figure 8D). The topology parameter analysis structure is listed in Table S5. There are 57 nodes (including 2 drugs, 26 potentially useful drug components, and 29 core targets) with 67 interaction edges in the network. From the network, we found there were seven ingredients that were highly associated with wound-healing targets.

#### 2.8.3. GO and KEGG Enrichment Analysis

**Figure 9:**
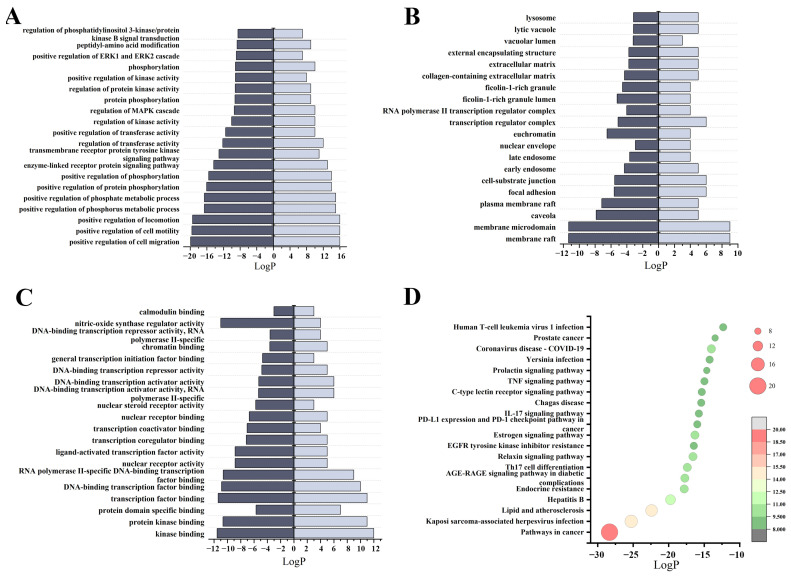
KEGG and GO enrichment results: (**A**) diagram of the top 20 biological process category terms based on GO BP enrichment analyses; (**B**) diagram of the top 20 cellular component category terms based on GO CC enrichment analyses; (**C**) diagram of the top 20 molecular function category terms based on GO MF enrichment analyses; (**D**) bubble plots of the top 20 pathways based on KEGG enrichment analyses.

The Metascape database was utilized to investigate the biological processes and KEGG pathways associated with the differential genes involved in wound healing [53] (Figure S8). In total, 786 entries with a significance threshold (*p* < 0.05) were identified through GO enrichment analysis. These entries comprised 679 biological process (BP) entries, 45 cellular component (CC) entries, and 62 molecular function (MF) entries. According to the GeneRatio value, the top 20 entries closely related to the disease were chosen for visualization. As shown in Figure 9A, BP mainly included the positive regulation of cell migration, positive regulation of cell motility, and positive regulation of locomotion. CC mainly contained membrane raft, membrane microdomain, and caveola (Figure 9B). MF is mainly related to kinase binding, protein kinase binding, and transcription factor binding (Figure 9C).

A total of 135 signaling pathways were obtained after KEGG Pathway analysis (*p* < 0.05). The top 20 pathways were filtered according to GeneRatio values for bubble map processing, including pathways in cancer, Kaposi sarcoma-associated herpesvirus infection, lipid and atherosclerosis, Endocrine resistance, AGE/RAGE signaling pathway in diabetic complications, Th-17 cell differentiation, IL-17 signaling pathway, Relaxin signaling pathway, C-type lectin receptor signaling pathway, EGFR tyrosine kinase inhibitor resistance, TNF signaling pathway, etc. (Figure 9D and Figure S8). The process of atherosclerosis regression is similar to wound healing [54]. During this regression of atherosclerosis, there is a decrease in neutrophil extracellular traps (NETs), which can cause tissue damage and hinder wound healing [55]. Furthermore, the key trigger for atherogenesis is the accumulation of low density lipoprotein and very low-density lipoprotein in the subendothelium, activating endothelial cells that recruit blood monocytes to infiltrate and differentiate into macrophages. These macrophages then uptake modified lipoproteins, forming foam cells and triggering a cascade of inflammatory responses. These suggest a connection between atherosclerosis and metabolic dysfunction, and chronic inflammatory processes, and regulating this pathway may have an important role for tissue injury and wound healing [56]. AGEs (Advanced Glycation End-products) are a type of molecules that result from the interaction of sugars with biological macromolecules like proteins and nucleic acids [57]. When AGEs bind to their receptor RAGE, they can trigger various signaling pathways, including NF-κB and MAPK, which can lead to negative effects such as inflammation, oxidative stress, dysfunction of vascular endothelial cells, and apoptosis [58]. Furthermore, the accumulation of AGEs in the skin is considered a potential risk factor for injuries and chronic ulcers [59]. Tumor necrosis factor (TNF), as a critical cytokine, can induce a wide range of intracellular signal pathways including apoptosis and cell survival as well as inflammation and immunity.

#### 2.8.4. Molecular Docking

**Figure 10:**
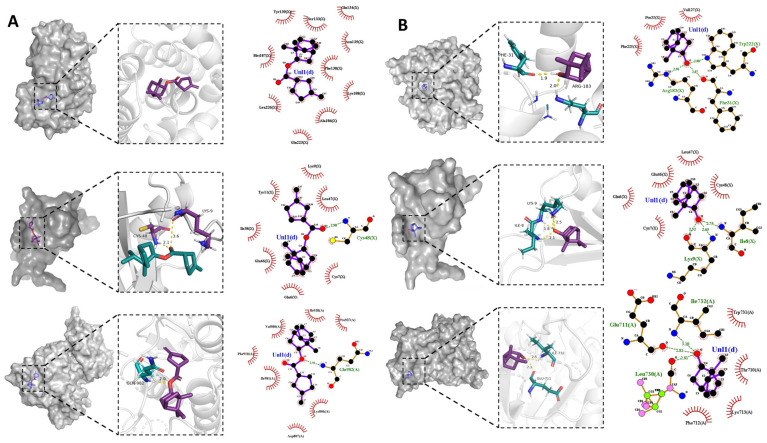
Molecular docking: (**A**) visualization of BBO27 docking with 3 target genes (AKT1, CXCL8, and EGFR); (**B**) visualization of L-borneol docking with 3 target genes (AKT1, CXCL8, and EGFR).

Through PPI analysis results and based on the literature, the top 5 gene targets (AKT1, CXCL8, EGFR, HIF1A, and JUN) (Table S5) were selected for molecular docking with the top 5 candidate components (Tetrahydrofuran-2-carboxylic acid (BBO28), 1,7,7-Trimethylbicyclo[2.2.1]heptan-2-yl-3-methylenecyclopentane-carboxylate(BBO27), Eugenol (BBO06), 4-Methoxy-3-tert-butylphenol (BBO09), Geranyl acetone (BBO52), and L-borneol) by the Autodock 4.2.6 software package (Table S6). Lower binding energy indicates a more stable ligand–receptor binding conformation and a higher likelihood of interaction. The results demonstrated that all candidate components and gene targets exhibited binding ability. Specifically, L-borneol and BBO27 exhibited low binding energy with five gene targets (Table S7). Binding energies below 0 indicated spontaneous binding of the ligand molecule to the receptor target, while binding energies below −5 kcal/mol indicated strong binding [60]. The binding energies of BBO27 and L-borneol with AKT1 (PDB code 5YVN), CXCL8 (PDB code 6N2U), and EGFR (PDB code 8A27) were all below −5 kal/mol, with values of −5.14, −5.11, −5.29, −5.42, −5.20, and −5.11 kal/mol, respectively. Their molecular docking diagram was presented in Figure 10. Based on the docking score and chemical bond distribution, AKT1, CXCL8, and EGFR may be the key targets of BBG-NEs in promoting wound healing.

### 2.9. Scratch Healing Assay

**Figure 11:**
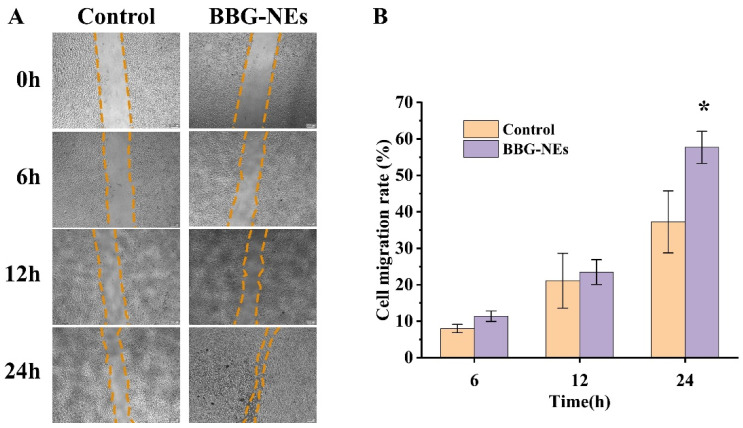
Cell scratch assay: (**A**) images of 0 h, 6 h, 12 h, and 24 h cell layer wounds showing the scratch width of BBG-NE effect on the migration ability of L929 cells; (**B**) degree of wound healing of BBG-NE-treated L929 cells. * *p* < 0.05, *n* = 3.

The GO enrichment results show that the first two biological processes in which BBG-NEs play a role in wound healing are the positive regulation of cell migration, and positive regulation of cell motility. To validate the results and determine the effect of BBG-NEs on the migration ability of L929 cells, we cultured L929 cells with BBG-NEs for 24 h. The results showed that the cell migration rate of the cells treated by BBG-NEs was greater than that of the control group from 6 h to 24 h (Figure 11A,B). These results also confirmed that BBG-NEs could effectively promote the migration of L929 cells.

### 2.10. Western Blotting

**Figure 12:**
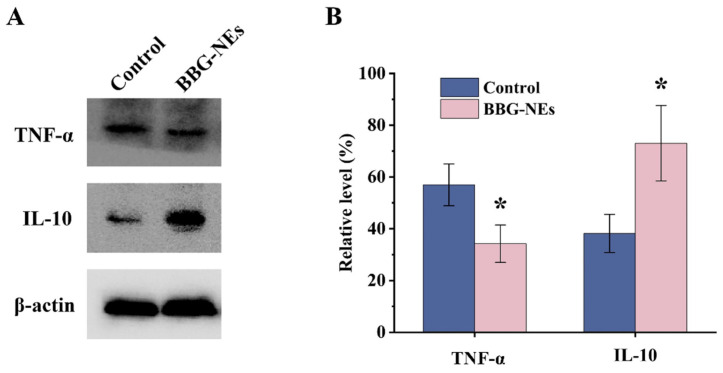
Western blotting experiments: (**A**) Western blotting was used to detect the expression levels of TNF-α and IL-10. (**B**) Relative expression levels of TNF-α and IL-10 in L929 cells treated with BBG-NES. β-actin was used as an internal reference; * *p* < 0.05, *n* = 3.

The expression levels of TNF-α and IL-10 proteins in L929 cells treated with BBG-NEs were detected using Western blotting (Figure 12A). Compared with the control group, the ratio of TNF-α gray value decreased in the experimental group. And the expression level of IL-10 in the experimental group was significantly higher than that in the control group, and the results of the statistical analysis of the data are shown in Figure 12B. The experimental results proved that the KEGG enrichment analysis was obtained to play a positive or negative role in the inflammatory response Kaposi sarcoma-associated herpesvirus infection, lipid and atherosclerosis, AGE/RAGE signaling pathway in diabetic complications, TNF signaling pathway, and other related pathways are important pathways involved in the process of BBG-NEs promoting wound healing.

## 3. Materials and Methods

### 3.1. Materials

BBO was purchased from Guizhou Miaoyao Biotechnology Co., Ltd. (Guizhou, China). L-borneol, ethylacetate, methyl salicylate were provided by Aladdin (Shanghai, China). BSP was obtained by Shaanxi Baichuan Biotechnology Co., Ltd. (Shaanxi, China); glycyrrhizic acid (purity > 98%) was bought from Xian Xiaocao Plant Technology Co., Ltd. (Shaanxi, China).

### 3.2. Optimization of the Prescription of BBG-NEs

The BBG-NEs were formulated using BBO, GA-BSP emulsifier, and distilled water. They were prepared by US method with a slight modification [25]. Briefly, BSP and GA were dissolved in distilled water as the aqueous phase, and the oil phase was added dropwise under stirring. After magnetic stirring for 20 min at room temperature, the coarse emulsion was then subjected to US using a JY92-IIN ultrasonic homogenizer (Ningbo Scientz Biotechnology Co., Ltd., Ningbo, China) that operated at 20 kHz with a maximum power output of 650 W. To obtain optimum and stable BBG-NEs, the effects of parameters on the appearance, particle size, and PDI of BBG-Nes were investigated systemically using single factor experiments. These parameters include BBO content (1%, 3%, 5%, 7%, 10%, *w*/*w*) (Table S8), BSP content (0.03%, 0.05%, 0.07%, 0.20%, 0.30%, *w*/*w*) (Table S9), ultrasonic time (2, 4, 6, 8, and 10 min) (Table S10) and ultrasonic power (65, 162.5, 195, 260, 357.5 W) (Table S11). An ice bath is used throughout the ultrasound process to prevent the loss of BBO.

### 3.3. Characterization of BBG-NEs

#### 3.3.1. Particle Size, PDI, Zeta Potential, and Morphological Observation

The mean particle size, PDI and zeta potential were detected by DelsaMax Pro (Beckman Coulter, Brea, CA, USA). The morphology of BBG-Nes was first observed by an optical microscope (XSP-C204, Chongqing Chongguang Industry Co., Ltd., Chongqing, China). And the transmission electron microscopy (TEM) images were obtained by a FEI Talos F200S instrument (FEI, Portland, OR, USA).

#### 3.3.2. Type Identification and pH

The type of BBG-NEs is determined by the diffusion rate of the fat-soluble dye Sudan red (red) and the water-soluble dye methylene blue (blue). If the diffusion rate of methylene blue is faster than that of Sudan red, it is classified as O/W type. Conversely, if the diffusion rate of Sudan red is faster than that of methylene blue, it is classified as W/O type. The pH of BBG-Nes was measured using a PHS-3E digital pH meter (Shanghai Yi Electrical Scientific Instruments Co., Ltd., Shanghai, China).

### 3.4. Stability Studies

#### 3.4.1. Long-Term Stability

The long-term stability of BBG-NEs was evaluated by storing it at room temperature for 90 days. The appearance was observed at 0 days and 90 days, and measurements of mean particle size, PDI, and zeta potential were conducted.

#### 3.4.2. Centrifugal Stability

To evaluate BBG-NEs’ centrifugal stability, they were centrifuged at 3000 rpm, 5000 rpm and 8000 rpm for 10 min, examining emulsion stratification and precipitation. Subsequently, particle size, PDI, and zeta potential measurements were conducted.

#### 3.4.3. Temperature Stability

The temperature stability of BBG-NEs was evaluated by exposing them to temperatures of 4 °C, 25 °C, and 40 °C for 48 h. Visual observations were made to assess any changes in appearance, while measurements of particle size, PDI, and zeta potential were conducted.

### 3.5. In Vitro Skin Permeation Study

The transdermal performance of BBG-NEs was investigated using L-borneol, the main active ingredient in BBO, as an indicator. Gas chromatography (Agilent Technologies Santa Clara, CA, USA) equipped with flame ionization detector (FID) and HP5 column (30 m × 0.32 mm × 0.5 μm) was utilized to determine the content of L-borneol in receiving solution. Nitrogen was used as the carrier gas. The temperature of oven was kept at 80 °C for 2 min, then increased to 100 °C at a rate of 5 °C/min, followed by a further increase to 200 °C at a rate of 20 °C/min and maintained for 4 min at this temperature. The injection inlet and detector temperatures were set at 220 °C and 240 °C, respectively. The injection volume was 0.6 μL [61].

The in vitro skin permeation studies were conducted on rat skin utilizing Franz diffusion cell (TP-6, Tianjin Shengda Sanhe Optical Instrument Co., Ltd., Tianjin, China) with a permeation area of 0.785 cm^2^. The rat skin was fixed between the donor and receptor compartments, with the dermis facing the receptor pool. The receiving solution was 20% ethanol-saline with continuous agitation (350 rpm) at 37 ± 0.5 °C. An amount of 2 mL of sample was placed in each donor compartment. At specific time intervals (1 h, 2 h, 4 h, 6 h, 8 h, 12 h, 24 h, 36 h, and 48 h), 1 mL samples were retrieved and subjected to gas chromatography for analysis. Cumulative L-borneol penetration per unit skin surface area (*Q_n_*, µg/cm^2^) was calculated using Equation (1):

(1) $$Q_{n}=(VC_{n}+\sum_{i=1}^{n-1}V_{i}C_{i})/A$$

where *V* and *V_i_* are the volume of the receiving cell (15 mL) and sampling volume, respectively, and *C_i_* and *C_n_* are the concentration of L-borneol in the receptor compartments at the ith sample and nth sample (mg/mL), respectively. *A* is the permeation area (cm^2^).

Permeation kinetics was analyzed using linear regression in four models: zero-order, first-order, Higuchi, and Ritger–Peppas. The model with the highest correlation coefficient (r) was selected as the best fit to describe the permeation profile.

### 3.6. Antioxidant Activity

The encapsulation effect on BBO’s antioxidant activity was evaluated using DPPH (1,1-Diphenyl-2-picrylhydrazine) and hydroxyl radical scavenging ability assay kits from Solarbio, Beijing, China. The experiment included five groups: BSP (group 1), GA (group 2), mixed emulsifier (GA + BSP, group 3), naked BBO (group 4), and BBG-NEs (group 5). Each group’s samples were diluted 5 times by the extraction solution in the kit for analysis.

#### 3.6.1. DPPH Free Radical Scavenging Activity

According to the DPPH (1,1-Diphenyl-2-picrylhydrazine) kit instructions [62], 10 µL of sample was added to 190 µL of DPPH ethanol solution and mixed evenly. The mixture was left to react in darkness at room temperature for 30 min. Then, the absorbance was measured at 515 nm using a microplate reader. The experiment was repeated triplicate and the *DPPH radical scavenging rate* was calculated using Equation (2):

(2) $$DPPH\;Free\;radical\;scavenging\;rate\left(\%\right)=(A_{c}-\left(A_{s}-A_{b}\right))/A_{c}\times 100\%$$

where *A_c_* is the absorbance of control (10 µL extraction solution + 190 DPPH), *A_s_* is the absorbance of the sample to be tested, and *A_b_* is the absorbance of samples’ color (10 µL sample + 190 µL ethanol).

#### 3.6.2. Hydroxyl Radical Scavenging Capacity

Hydroxyl radical (•OH) is formed through the Fenton reaction when H_2_O_2_ is present along with Fe^2+^. The scavenging activity of Hydroxyl radical (•OH) was evaluated by measuring the inhibition of Fe^2+^ oxidation as instructed in the hydroxyl radical scavenging kit. Briefly, 50 µL of the sample was incubated with 300 µL of the reagents in the kit for 60 min at 37 °C. The reaction mixture was then centrifuged at 10,000 rpm for 10 min, and the absorbance of the supernatant was measured at 536 nm. The hydroxyl radical scavenging capacity is calculated using Equation (3).

(3) $$Hydroxyl\;radical\;scavenging\;rate\left(\%\right)=\left(A_{s}-A_{c}\right)/\left(A_{b}-A_{c}\right)\times 100\%$$

where *A_b_* is the absorbance of blank tube; *A_c_* is the absorbance of control tube; and *A_s_* is the absorbance of sample tube.

### 3.7. Cytotoxicity Assays

The cytotoxic effects of BBG-NEs on fibroblast cells were investigated using the MTT assay. L929 fibroblast cells were maintained in RPIM-1640 medium supplemented with 10% fetal bovine serum and 1% penicillin-streptomycin in a 5% CO_2_ incubator at 37 °C. The cells were seeded into 96-well plates at a density of 5 × 10^3^ cells/well and incubated at 37 °C. After 24 h of incubation, the medium was refreshed with fresh medium containing different concentrations of BBG-NEs and incubated for another 24 hours. Subsequently, 20 µL of a 5 mg/mL MTT solution was added to each well and the cells were incubated for 4 h. Following this, 100 µL of DMSO was introduced into each well, and the mixture was shielded from light for 10 min. Thereafter, the absorbance at 560 nm was determined.

### 3.8. Wound-Healing Assessment

The animal experiments were approved by the Laboratory Animal Welfare and Ethics Review Committee of Guizhou University of Traditional Chinese Medicine (No. GZY20231228002). Sprague Dawley (SD) rats were first anesthetized and then a full-thickness circular skin with a diameter of 1 cm on the dorsal area of rats was cut off to create a wound. The wound was subsequently sterilized using iodophor, and the initial dose of treatment was administered after a 30 min interval following the trauma. The wounds were then randomly divided into four groups: control group, treated with saline once daily; BBO group, treated with BBO once daily; emulsifier group, treated with emulsifier (0.3% GA and 0.07% BSP aqueous solution) once daily; BBG-NEs group, treated with BBG-NEs once daily. An amount of 0.1 mL of each treatment was applied topically, and the wound-healing was observed daily. Wounds were photographed at days 0, 1, 3, 7, and 14, and the areas were calculated using the Image-J 1.54d software package. The percentage of wound-healing rate [63] was calculated using Equation (4):

(4) $$Wound\;healing\;rate\left(\%\right)={(S}_{A}-S_{i})/S_{A}\times 100\%$$

where *S_A_* is the initial wound area, and *S_i_* is the specific day wound area.

### 3.9. Network Pharmacology

#### 3.9.1. Screening of Active Ingredients in BBG-NEs

The chemical compositions of BBO and BSP were determined by searching the Pubmed database (https://pubmed.ncbi.nlm.nih.gov (accessed on 10 May 2023)). These components were then screened in the swissADME database (http://www.swissadme.ch/ (accessed on 19 May 2023)) for pharmacokinetic activity and drug similarities. Components with high GI absorption and DrugLikeness with two or more Yes qualities were retained [64]. The remaining components were subjected to target prediction using the SwissTargetPrediction database (http://www.SwissTargetPrediction.ch/ (accessed on 25 May 2023)), recording targets with a probability above 0.0.

#### 3.9.2. Wound-Healing-Related Target Screening

Wound-healing-related targets were obtained by searching the GeneCards (https://www.genecards.org/ (accessed on 10 June 2023)) and Online Mendelian Inheritance in Man (OMIM) (https://omim.org/ (accessed on 10 June 2023)) databases using the keyword “wound healing”. Genes with correlation scores > 2.0 in GeneCards were combined with OMIM database genes, while removing the overlapping results.

#### 3.9.3. Construction of Protein–Protein Interaction (PPI) Network

To explore the interaction between target proteins, we intersected the obtained drug targets with genes associated with wound healing and identified overlapping targets. The gene symbols of these overlapping targets were then inputted into the online platform STRING (http://string-db.org (accessed on 7 July 2023)) to gather protein–protein interaction (PPI) network data, focusing on the organism “Homo sapiens”. Additionally, the Cytoscape 3.9.1 software was utilized for the analysis and visualization of the PPI network. Only genes meeting the criteria of betweenness unDir > 119.6, closeness unDir > 0.00396, and degree unDir > 30.66 were selected and visualized within the PPI network.

#### 3.9.4. GO and KEGG Enrichment Analysis

Gene ontology (GO) and Kyoto Encyclopedia of Genes and Genomes (KEGG) pathway enrichment analysis were conducting by connecting targets to the Metascape database (https://metascape.org (accessed on 13 July 2023)). *p*-values obtained from the Metascape database were assigned with a significance threshold below 0.05. GO results were presented as bar graphs and KEGG results were presented as bubble graphs.

#### 3.9.5. Molecular Docking

The compounds with the top 5 values in BBG-NE active ingredient–target network were selected as ligands. In addition, L-borneol were included based on the literature [61]. The three-dimensional structure of these compounds was obtained from the PubChem database. The targets with the top 5 value were selected in the drug–disease common target PPI network. The protein sequences and structures were obtained from Protein Data Bank (PDB) (http://www.rcsb.org/pdb/ (accessed on 29 July 2023)). AutoDock tool was used to remove the water molecules from the protein crystal structure and add the hydrogen atoms. Molecular docking was conducted using AutoDock 4.2.6 to obtain the docking affinity of the active ingredient to the key target (affinity, kcal/mol). Lastly, molecular docking results were visualized using PyMOL 3.8 software.

### 3.10. Scratch Healing Assay

Fibroblast L929 cells were collected and made into a cell suspension, which was added to well plates at a concentration of approximately 1 × 10^5^ cells/well and incubated overnight at 37 °C under 5% CO_2_. After the cells reached 90% fusion, the center of each well monolayer of cells was crossed with the tip of a 10 µL pipette. Cell debris was removed with PBS, and L929 cells were treated with BBG-NEs. Each scratch wound was observed microscopically at 0, 6, 12, and 24 h, and three randomly selected fields of view (×100) were used to assess cell migration ability.

### 3.11. Western Blotting

L929 cells were collected after 24 h of treatment with BBG-NEs, lysed, centrifuged at 4 °C for 10 min, and stored at −80 °C until use. Total proteins were separated by 10% SDS-PAGE, and the proteins in the gel were transferred to a PVDF membrane. Proteins were then incubated overnight at 4 °C with primary antibodies (including TNF-α and IL-10). Then, proteins were incubated with secondary antibody at room temperature for 1 h. Immunoreactive protein bands were imaged using a fully automated chemiluminescence image analysis system with β-actin as an internal reference. Finally, quantitative analysis was performed using the ImageJ software package to determine the gray scale values of the protein bands.

## 4. Conclusions

This research yielded stable, well-distributed multifunctional novel NEs (BBG-NEs) via US method with BSP and GA as natural emulsifiers and BBO as natural oil phase. The BBG-NEs had a particle size of 170.2 ± 2.39 nm and a PDI of 0.212 ± 0.008. NE encapsulation significantly improved the transcutaneous permeation of BBO and enhanced its DPPH and hydroxyl radical scavenging abilities. Interestingly, BBG-NEs exhibited better wound-healing-promoting effect compared with naked BBOs and emulsifiers. After 14 days of treatment, rat wounds were basically completely healed, with intact hair growth around the wounds and almost restored pre-traumatic morphology. Network pharmacology and molecular docking results showed that 26 core components 29 potential targets were screened out. BBG-NEs may promote wound healing by affecting IL-17, AGE-AGEs, and lipid and atherosclerosis signaling pathways as well as pivotal targets like AKT1, CXCL8, and EGFR. Cell scratch and Western blotting assays showed that BBG-NEs had a migration-promoting effect on fibroblast L929 cells, as well as inhibiting the expression of TNF-α and promoting the expression of the anti-inflammatory factor IL-10. However, validation experiments of the network pharmacology and molecular docking results still need to be further investigated. This study can offer novel insights and practical guidance for the development of innovative multi-functional NEs utilizing natural emulsifiers.

## Figures and Tables

**Table 1:** Results of fitting BBO-NE to different release curves.

Release Model	Regression Equation	Correlation Coefficient (r)
Zero-order	*Q* = 0.15*t* − 0.27	0.994
First-order	*Q* = −44.22 [1 − exp(−0.003*t*)]	0.991
Higuchi	*Q* = 1.219*t*^1/2^ − 2.05	0.972
Ritger–Peppas	*Q* = 0.103*t*^1.1^	0.993

## Data Availability

Data are contained within the article.

## Supplementary Materials

The following supporting information can be downloaded at: https://www.mdpi.com/article/10.3390/molecules29091994/s1, Figure S1: Effect of oil phase content on BBG-NEs; Figure S2: Effect of Bletilla striata polysaccharide content on BBG-NEs; Figure S3: Effect of ultrasonic time on BBG-NEs; Figure S4: Effect of ultrasonic power on BBG-NEs; Figure S5: Centrifugal stability of BBG-NEs; Figure S6: Temperature stability of BBG-NEs; Figure S7: Transdermal comparison of BBG-NEs and BBO; Figure S8: Ingredient–target–pathway network; Table S1: Particle size, PDI, and potential of BBG-NEs prepared by ultrasonication with different BBO content; Table S2: Particle size, PDI, and potential of BBG-NEs prepared by ultrasonication with different content of BSP; Table S3: Particle size, PDI and potential of BBG-NEs prepared by ultrasonication with different ultrasonic time; Table S4: Particle size, PDI, and potential of BBG-NEs prepared by ultrasonication with different ultrasonic power; Table S5: Parameters of genes associated with BBG-NEs for wound healing (top 5); Table S6: Compounds in the drug–ingredient–target network (top 5); Table S7: The molecular docking results (top 5). Table S8: Nanoemulsion formulation with different BBO content. Table S9: Nanoemulsion formulation with different content of BSP. Table S10: Nanoemulsion formulation with different ultrasonic time. Table S11: Nanoemulsion formulation with different ultrasonic power.
