# Supplementary material for: Green Synthesis of Blumea balsamifera Oil Nanoemulsions Stabilized by Natural Emulsifiers and Its Effect on Wound Healing
Source: Molecules. 2024 Apr 26;29(9):1994. doi: 10.3390/molecules29091994 (PMC11085480; doi:10.3390/molecules29091994)
Supplement: Supplementary file 1 [file molecules-29-01994-s001.zip › molecules-2923854-supplementary.pdf]

Green synthesis of *Blumea balsamifera* oil nanoemulsions stabilized by natural emulsifiers and its effect on wound healing

Lingfeng Du[1], Chunfang Ma[1], Bingnan Liu[1], Wei Liu[1], Yue Zhu[2,3], Zuhua Wang[2,3], Teng Chen[2,3*], Luqi Huang[4*], Yuxin Pang[1,2,5*]

[1] College of Chinese Medicine Resources, Guangdong Pharmaceutical University, Yunfu 527325, China

[2] College of Pharmaceutical Sciences, Guizhou University of Traditional Chinese Medicine, Guiyang 550025, China;

[3] Nano-Drug Technology Research Center, Guizhou University of Traditional Chinese Medicine, Guiyang 550025, China

[4] China Academy of Chinese Medical Sciences, Beijing 100700, China

[5] Yunfu Traditional Chinese Medicine Resources and Germplasm Resources Database Management Center, Yunfu 527325, China

*Correspondence: **Teng Chen**, chenteng0907@163.com; **Luqi Huang**, huangluqi01@126.com; **Yuxin Pang**, pyxmarx@126.com

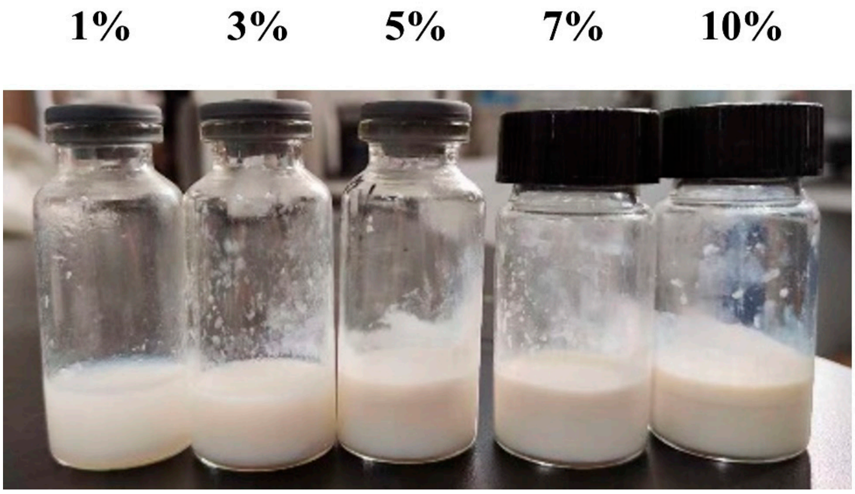

Figure S1. Effect of oil phase content on BBG-NEs.

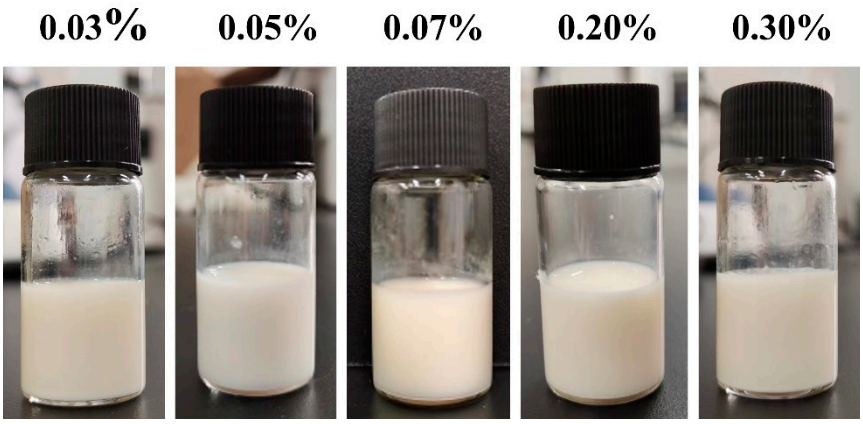

Figure S2. Effect of *Bletilla striata* polysaccharide content on BBG-NEs.

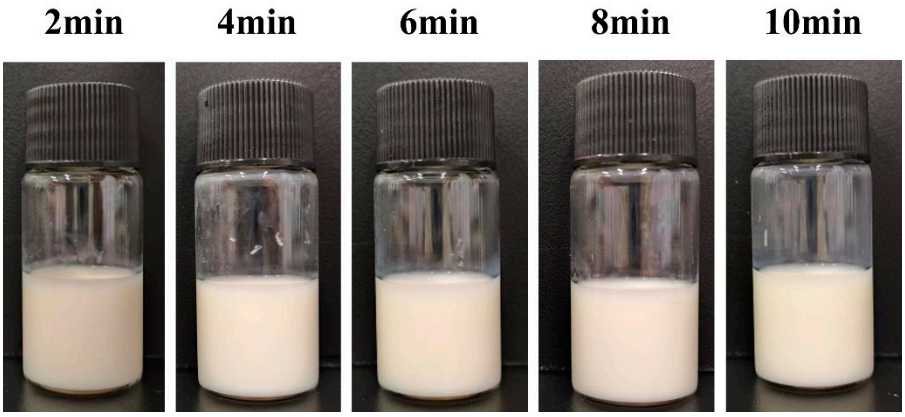

Figure S3. Effect of ultrasonic time on BBG-NEs.

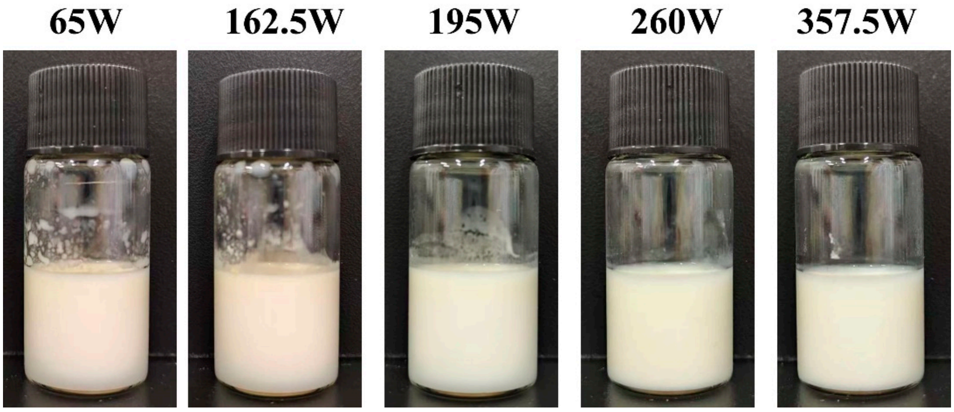

Figure S4. Effect of ultrasonic power on BBG-NEs.

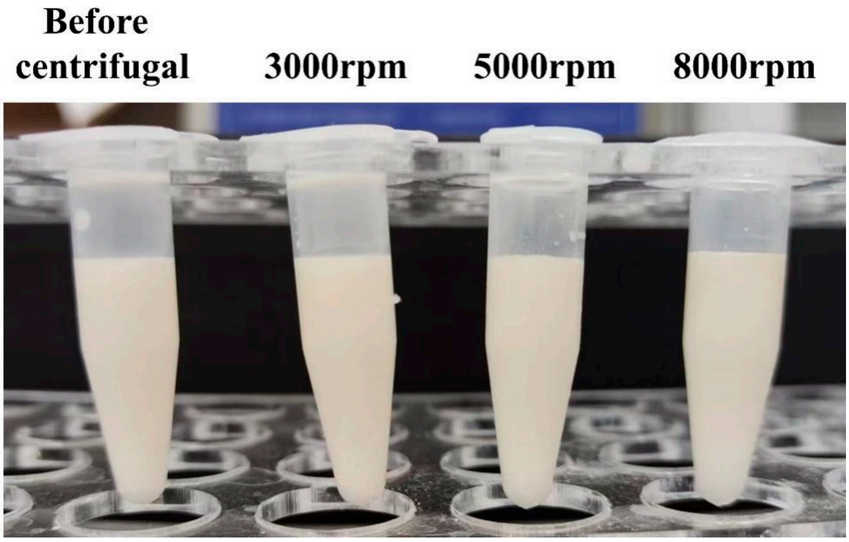

Figure S5. Centrifugal stability of BBG-NEs.

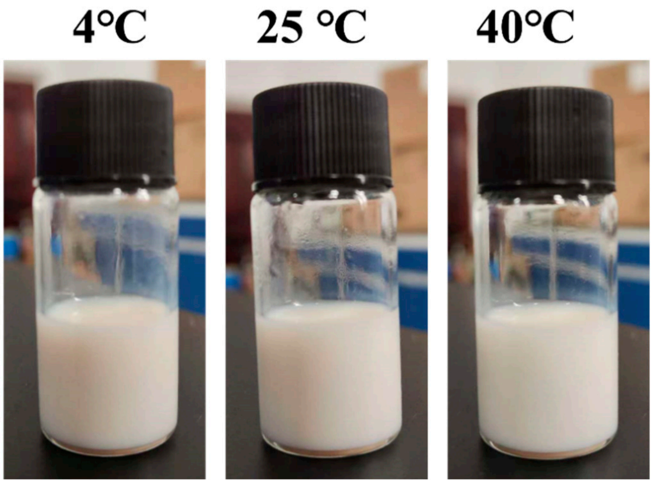

Figure S6. Temperature stability of BBG-NEs.

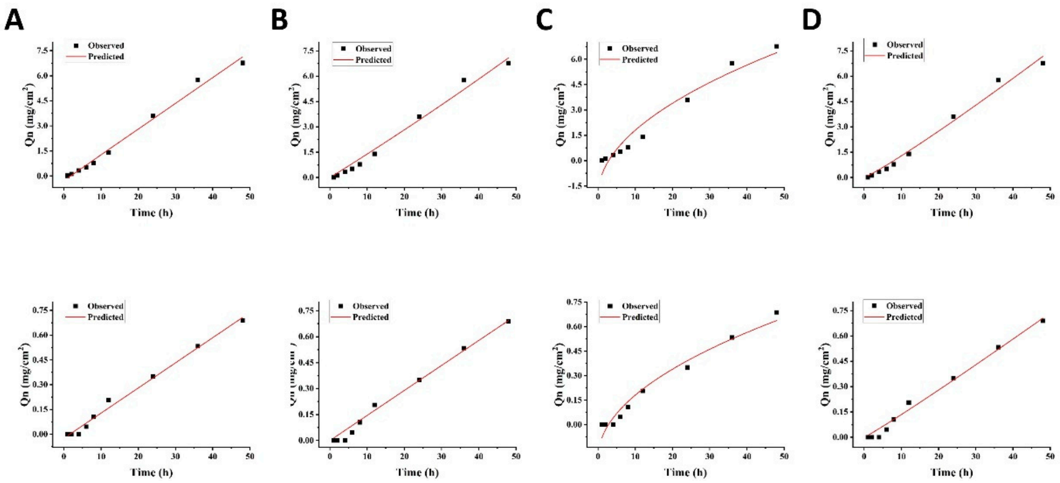

Figure S7. Transdermal comparison of BBG-NEs (upper) and BBO (lower): (A) Zero-order; (B) First-order; (C) Higuchi; (D) Ritger-peppas.

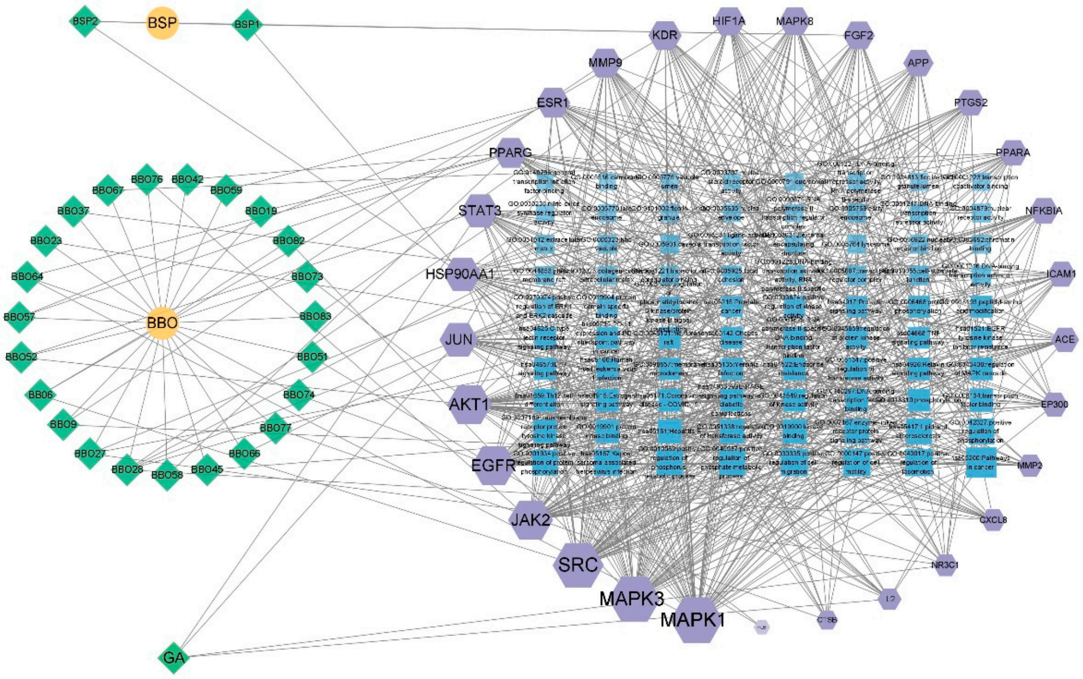

Figure S8. Ingredient-target-pathway network, with purple hexagonal nodes representing the targets, the blue quadrilateral nodes represent pathways, and green diamond-shaped nodes represent ingredients in the formulation. The larger the area of the node graph and the darker the color

represent the larger the DEGREE value of the component.

Table S1. Particle size, PDI and potential of BBG-NEs prepared by ultrasonication with different BBO content.

No.	Oil phase (BBO), w/w	Appearance	Zeta potential (mV)	Particle size(nm)	PDI
1	1%	Homogeneous milky white liquid, opaque	-25.48±1.69	222.2±7.06	0.243±0.013
2	3%	Homogeneous milky white liquid, opaque	-55.98±13.58	229.71±6.82	0.238±0.001
3	5%	Milky white liquid, layered	——	——	——
4	7%	Milky white liquid, layered	——	——	——
5	10%	Milky white liquid, layered	——	——	——

Table S2. Particle size, PDI and potential of BBG-NEs prepared by ultrasonication with different content of BSP.

No.	Emulsifier (BSP), w/w	Appearance	Zeta potential (mV)	Particle size(nm)	PDI
1	0.03%	Homogeneous milky white liquid, opaque	-41.70±1.52	200.39±2.17	0.238±0.001
2	0.05%	Homogeneous milky white liquid, opaque	-30.17±0.72	213.95±5.89	0.238±0.002
3	0.07%	Homogeneous milky white liquid, opaque	-38.37±0.94	209.74±3.61	0.241±0.005
4	0.2%	Homogeneous milky white liquid, opaque	-48.28±10.35	221.59±2.22	0.268±0.039
5	0.3%	Homogeneous milky white liquid, opaque	-32.94±0.53	212.66±3.51	0.306±0.044

Table S3. Particle size, PDI and potential of BBG-NEs prepared by ultrasonication with different ultrasonic time.

No.	Ultrasonic time, min	Appearance	Zeta potential	Particle size(nm)	PDI
1	2min	Homogeneous milky white liquid, opaque	-38.37±0.94	209.74±3.61	0.241±0.005
2	4min	Homogeneous milky white liquid, opaque	-35.23±0.81	177.04±3.05	0.231±0.007
3	6min	Homogeneous milky white liquid, opaque	-32.86±0.71	170.2±2.39	0.212±0.008
4	8min	Homogeneous milky white liquid, opaque	-27.51±0.99	194.11±8.34	0.281±0.056
5	10min	Homogeneous milky white liquid, opaque	-32.23±3.13	162.24±3.99	0.189±0.018

Table S4. Particle size, PDI and potential of BBG-NEs prepared by ultrasonication with different ultrasonic power.

No.	Ultrasonic power , W	Appearance	Zeta potential	Particle size(nm)	PDI
1	65	Homogeneous milky white liquid, opaque, with oil droplets on the surface	-22.18±0.57	268.06±6.95	——
2	162.5	Homogeneous milky white liquid, opaque	-32.86±0.71	170.2±2.39	0.212±0.008
3	195	Homogeneous milky white liquid, opaque	-34.63±1.63	177.85±5.96	0.209±0.015
4	260	Homogeneous milky white liquid, opaque	-32.22±1.99	173±2.97	0.214±0.012
5	357.5	Homogeneous milky white liquid, opaque	-29.69±2.05	143.16±3.463	0.207±0.036

Table S5. Parameters of genes associated with BBG-NEs for wound healing (top 5).

Protein names	Gene names	Betweenness unDir	Closeness unDir	Degree unDir
Serine/Threonine Kinase 1	AKT1	601.5349403	0.005617978	98
C-X-C Motif Chemokine Ligand 8	CXCL8	529.9941482	0.004901961	73
Epidermal Growth Factor Receptor	EGFR	206.7625745	0.005263158	86
Hypoxia Inducible Factor 1 Subunit Alpha	HIF1A	399.3425545	0.005154639	82
Jun Proto-Oncogene, AP-1 Transcription Factor Subunit	JUN	262.9061061	0.005050505	78

Table S6. Compounds in the drug-ingredient-target network (top 5).

Compound name	Molecular function	2D structure	Degree
Tetrahydrofuran-2-carboxylic acid	$C_5H_8O_3$	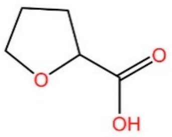	6
1,7,7-Trimethylbicyclo[2.2.1]heptan-2-yl-3-methylenecyclopentane-carboxylate	$C_{17}H_{26}O_2$	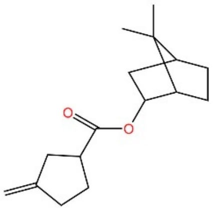	4
Eugenol	$C_{10}H_{12}O_2$	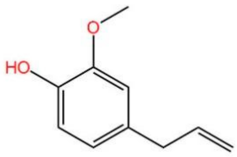	3

4-Methoxy-3-tert-butylphenol	$C_{11}H_{16}O_2$	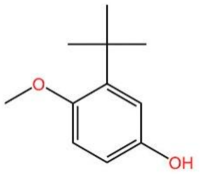	3
Geranyl acetone	$C_{13}H_{22}O$	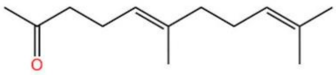	3
L-borneol	$C_{10}H_{18}O$	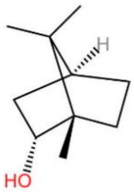	/

Table S7. The molecular docking results (top 5).

Protein<br>Affinity kcal/mol<br>Compound	AKT (PDB code 5YVN)	CXCL8 (PDB code 6N2U)	EGFR (PDB code 8A27)	HIF1A (PDB code 8HE3)	JUN (PDB code 7NYO)
1,7,7-trimethylbicyclo[2.2.1]heptan-2-yl-3-methylenecyclopentane-carboxylate	-5.42	-5.20	-5.11	-3.83	-4.35
Tetrahydrofuran-2-carboxylic acid	-4.12	-3.80	-3.5	-3.23	-2.41
Eugenol	-3.61	-4.26	-4.35	-2.45	-2.19
4-Methoxy-3-tert-butylphenol	-3.86	-4.07	-3.41	-3.07	-2.62
Geranyl acetone	-4.21	-4.50	-3.22	-2.73	-2.68
L-borneol	-5.14	-5.11	-5.29	-4.2	-3.7

Table S8. Nanoemulsion formulation with different BBO content.

No.	Oil phase (BBO), w/w	Emulsifier (polysaccharide), w/w	Co-emulsifier (glycyrrhizic acid), w/w	Ultrasonic time, min	Ultrasonic power, W
1	1%	1%	0.3%	2	162.5
2	3%	1%	0.3%	2	162.5
3	5%	1%	0.3%	2	162.5
4	7%	1%	0.3%	2	162.5
5	10%	1%	0.3%	2	162.5

Table S9. Nanoemulsion formulation with different content of BSP.

No.	Oil phase (BBO), w/w	Emulsifier (polysaccharide), w/w	Co-emulsifier (glycyrrhizic acid), w/w	Ultrasonic time, min	Ultrasonic power, W
1	3%	0.03%	0.3%	2	162.5
2	3%	0.05%	0.3%	2	162.5
3	3%	0.07%	0.3%	2	162.5
4	3%	0.2%	0.3%	2	162.5
5	3%	0.3%	0.3%	2	162.5

Table S10. Nanoemulsion formulation with different ultrasonic time.

No.	Oil phase (BBO), w/w	Emulsifier (polysaccharide), w/w	Co-emulsifier (glycyrrhizic acid), w/w	Ultrasonic time, min	Ultrasonic power, W
1	3%	0.07%	0.3%	2	162.5
2	3%	0.07%	0.3%	4	162.5
3	3%	0.07%	0.3%	6	162.5
4	3%	0.07%	0.3%	8	162.5
5	3%	0.07%	0.3%	10	162.5

Table S11. Nanoemulsion formulation with different ultrasonic power.

No.	Oil phase (BBO), w/w	Emulsifier (polysaccharide), w/w	Co-emulsifier (glycyrrhizic acid), w/w	Ultrasonic time, min	Ultrasonic power, W
1	3%	0.07%	0.3%	6	65
2	3%	0.07%	0.3%	6	162.5
3	3%	0.07%	0.3%	6	195
4	3%	0.07%	0.3%	6	260
5	3%	0.07%	0.3%	6	357.5
